# Salidroside Protects Dopaminergic Neurons by Preserving Complex I Activity via DJ-1/Nrf2-Mediated Antioxidant Pathway

**DOI:** 10.1155/2019/6073496

**Published:** 2019-05-16

**Authors:** Ruru Li, Songhai Wang, Tao Li, Leitao Wu, Yan Fang, Yang Feng, Lingling Zhang, Jianzong Chen, Xin Wang

**Affiliations:** ^1^Department of Chinese Medicine, Xijing Hospital, Fourth Military Medical University, Xi'an 710032, China; ^2^Department of Toxicology, Faculty of Preventive Medicine, Fourth Military Medical University, Xi'an 710032, China

## Abstract

The pathogenic mechanism of Parkinson's disease (PD) remains to be elucidated; however, mitochondrial dysfunction at the level of complex I and oxidative stress is suggestively involved in the development of PD. In our previous work, salidroside (Sal), an active component extracted from the medicinal plant *Rhodiola rosea* L., might protect dopaminergic (DA) neurons through modulating ROS–NO-related pathway. However, the mechanism of Sal-induced neuroprotective effects against PD remains poorly understood. Therefore, we further investigated whether Sal plays neuroprotective effects by activating complex I via DJ-1/Nrf2-mediated antioxidant pathway. The results showed that Sal remarkably attenuated MPP^+^/MPTP-induced decline in cell viability, accompanied by decreases in reactive oxygen species (ROS), malondialdehyde (MDA), and 8-hydroxy-deoxyguanosine (8-OHdG) contents and increases in the superoxide dismutase (SOD), catalase (CAT), and glutathione peroxidase (GSH-Px), as well as glutathione (GSH) levels. Furthermore, Sal greatly improved the behavioral performance and prevented the severe reduction of TH-positive neuron numbers in the substantia nigra (SN). Moreover, in comparison with the MPP^+^/MPTP group, Sal increased the nuclear translocation of DJ-1 and Nrf2 and the mitochondrial translocation of DJ-1, accompanied by activating complex I. Furthermore, silencing of DJ-1/Nrf2 inhibited the increase of complex I activity and cell viability elicited by Sal. Together, these results support the neuroprotective effect of Sal against MPP^+^/MPTP-induced DA neurons damage.

## 1. Introduction

Parkinson's disease (PD) is a prevalent neurodegenerative movement disorder affecting 2%-3% of the population above the age of 65 worldwide [1, 2]. The hallmark of PD is the degeneration of dopaminergic (DA) neurons in the substantia nigra (SN) and the presence of Lewy bodies (LBs) [3]. The etiology of PD remains unclear, but extensive evidences from postmortem brain tissues have suggested that oxidative stress and deficiency of complex I activity are related to the pathogenic mechanism of PD [4, 5].

Oxidative stress, which occurs mainly due to overproduction of reactive oxygen species (ROS), activates a cascade of events leading to DA neuron degeneration [6]. Mitochondrial complex I is the major site where ROS generate, and once damaged, the mitochondria complex I would not only produce more ROS but also become the target for oxidative stress damage [7]. Complex I dysfunction can result in excess generation of ROS, which in turn will lead to the oxidative modification of complex I in a positive feedback loop to exacerbate complex I dysfunction [8]. Given the role of complex I in generating ROS and undergoing oxidative modification damage, it is a promising target for antioxidant therapeutic strategies.

Recently, researchers have focused on the pharmacological targeting of antioxidant gene transcription, including DJ-1 (gene: *PARK7*) and nuclear factor erythroid-2-related factor 2 (protein: Nrf2; gene: *NFE2L2*), attempting to inhibit oxidative stress and deactivation of complex I and limit neuronal damage [9, 10]. DJ-1 is known to act as an antioxidant via directly scavenging ROS and upregulating the expression of other antioxidant genes [11]. One of the most relevant target genes is the transcriptional factor Nrf2, a master regulator of antioxidant gene, which activates a range of antioxidant enzymes via antioxidant response element (ARE) enhancer [11].

*Rhodiola rosea* L. has been used for centuries in traditional medicinal practices in Asia, Scandinavia, and Eastern Europe to stimulate nerves, enhance physical and mental performance, improve resistance to high-altitude sickness, and treat fatigue, psychological stress, and depression [12, 13]. Salidroside (Sal), an active component extracted from *Rhodiola rosea* L., possesses multiple pharmacological properties, including antioxidant, antiaging, and antifatigue [12].

Our previous study has shown Sal plays neuroprotective effect through modulating the ROS–NO-related pathway [14]. However, the effects of Sal on complex I dysfunction, accompanied by neuronal loss induced by oxidative stress, and the protective mechanism of Sal against MPTP/MPP^+^ remains unclear. In light of these findings, the present study was designed to elucidate whether Sal plays neuroprotective effect through activating complex I via DJ-1/Nrf2 -elated antioxidant pathway in the MPTP/MPP^+^-induced PD model.

## 2. Materials and Methods

### 2.1. Cell Culture and Treatment

MN9D cells, a DA neuronal cell line derived from mouse mesencephalon, were purchased from Type Culture Collection of the Chinese Academy of Sciences (Shanghai, China). The cells were cultured in RPMI 1640 medium (Hyclone, Logan, USA) with 10% FBS (Gibco, Gaithersburg, MD) and maintained at 37°C in a 5% CO_2_ atmosphere. The cells were pretreated with Sal (10, 25, and 50 *μ*M) for 24 h and incubated with 200 *μ*M MPP^+^ for an additional 24 h. The cells were divided into the following groups: control group, MPP^+^ group, Sal group, and groups of different concentrations of Sal before MPP^+^ treatment.

### 2.2. Cell Transfection

siRNA duplexes were synthesized and purified by Biomics Biotechnologies (Nantong, China). SiRNA was diluted to 50 nm with Opti-MEM. The siRNA sequences of *NFE2L2* and *PARK7* are depicted in Table 1. The transfection was performed using Lipofectamine 2000 (Thermo Fisher, Waltham, MA, USA). After 72 h, the transfection efficiency was detected by western blot.

### 2.3. Cell Viability

Cell viability was measured using MTT assay kit (Sigma, MO, USA), following the manufacturer's instructions. The absorbance at 570 nm was measured, and cell viability was expressed as the percentage to the control group.

### 2.4. Animals and Drug Treatments

Adult male C57BL/6 mice (6–8 weeks) weighing 22–25 g were treated and maintained according to the guidelines established by the National Institutes of Health for the care and use of laboratory animals and were approved by the Animal Care Committee of the Fourth Military Medical University and Chinese Academy of Medical Sciences. Mice were randomly assigned to six groups (10 mice per group) including Group A, control mice; Group B, MPTP challenged; Group C, MPTP challenged and then Sal treated (15 mg/kg); Group D, MPTP challenged and then Sal treated (50 mg/kg); Group E, Sal treated alone (15 mg/kg); and Group F, Sal treated alone (50 mg/kg). Mice received intraperitoneal injection with MPTP for 5 consecutive days (30 mg/kg/day). Control mice and Sal alone-treated mice received intraperitoneal injection of saline for 5 days. After MPTP injection, the mice of the last four groups received intraperitoneal injection of Sal for 7 days, and the control and MPTP groups received an equivalent volume of saline. Seven days after the last administration of MPTP or saline, mice were subjected to behavioral tests.

### 2.5. Behavioral Tests

The behavior performance was analyzed by the pole and open field tests as previously described [15]. In brief, the pole test was performed to detect the impairment of limb movement [16]. After placing the mice on a rough pole, the time for the mice to turn completely downwards (T-turn) and climb down to the floor (T-LA) was recorded. Open field is a popular model for assessing spontaneous movement activity and anxiety-like behavior [17–19]. The mice were placed in a wooden box (40 × 40 cm^2^) that was enclosed by 10 cm high walls. The brown floor with a pattern of 16 squares was marked with black lines. Animals were placed in the rear left square and allowed to freely explore the field for 5 min. The speed was recorded to measure the locomotion level.

### 2.6. Measurement of ROS

The levels of ROS were measured by Reactive Oxygen Species Assay Kit (Beyotime, Beijing, China) as previously described [14]. And, the fluorescence intensities were analyzed using a microplate reader. The measured fluorescence values were expressed as a percentage of the fluorescence of the control group.

### 2.7. Measurement of Complex I Activity

The complex I activity was measured by the Complex I Enzyme Activity Microplate Assay Kit (Abcam, MA, USA), following the manufacturer's instructions, and OD values at 450 nm was recorded in the kinetic mode at room temperature (RT) for 30 min. The complex I activity was expressed as the change in absorbance per minute per amount of sample loaded into the well.

### 2.8. Immunofluorescent Assay

For immunofluorescent assay, the SN of brains were isolated, frozen, and cut into 30 µm slices on a Cryostats (Thermo Fisher, Waltham, MA, USA). Then, the slides were permeabilized and blocked in 2% BSA, incubated with TH antibody (Sigma-Aldrich, USA) overnight, and treated with Cy3-labeled goat anti-chicken IgG for 1 h in the dark. Images were analyzed by using an inverted microscope (IX51-12PH, Olympus) at a magnification of 200×.

### 2.9. Western Blot

The total protein was lysed in RIPA lysis, the nuclear and cytoplasmic proteins were extracted with the Nuclear and Cytoplasmic Protein Extraction Kit, and the mitochondrial protein in the cell or SN was extracted with the Cell Mitochondria Isolation Kit or Tissue Mitochondria Isolation Kit (Beyotime, Beijing, China). Equal quantity of proteins were electrophoresed on SDS-PAGE gel and transferred to PVDF membranes. The membranes were blocked and incubated with SOD1 (CST, USA), SOD2 (CST), GSH-Px (CST), DJ-1(CST), Nrf2 (Sigma-Aldrich), and Keap1 (Sigma-Aldrich) antibodies at 4°C overnight followed by goat anti-rabbit IgG antibody (Santa Cruz Biotechnology). The membrane was visualized using chemiluminescent reagents, and the protein level was quantified by using Image J. All protein levels were adjusted for the corresponding *β*-actin (CST), histone H3 (CST), and VDAC1 (CST) and were consistent across different treatment conditions.

### 2.10. Measurement of MDA, 8-OHdG, GSH, GSH-Px, Cu/Zn-SOD (SOD1), Mn-SOD (SOD2), and CAT in SN

To extract the protein, SN was homogenized in Tris buffer (10 mM Tris-HCl, 1 mM EDTA, 0.32 M sucrose, and pH 7.8) as 10% (w/v) using a Teflon homogenizer (Glas-Col homogenizer system, Vernon Hills, USA), centrifuged at 20,000 × g for 10 min. The MDA, GSH, GSH-Px, SOD1, SOD2, and CAT contents in the SN were measured by using a commercially available kit (Jiancheng Bioengineering Institute, Nanjing, China) following the manufacturer's instructions. The 8-OHdG level was measured using 8-hydroxy 2-deoxyguanosine ELISA kit (Abcam, MA, USA), according to the manufacturer's protocol. The OD values were measured using a microplate reader and were expressed as percentage of the OD values of the control group.

### 2.11. Statistical Analysis

Quantitative data were reported as mean ± standard deviation of three independent experiments. Statistical analysis among different groups was performed with one-way analysis of variance (ANOVA), followed by Tukey's multiple comparison test as post hoc. A value of *P* < 0.05 was considered significant.

## 3. Results

### 3.1. Effect of Sal on MPP^+^-Induced Reduction of Cell Viability

**Figure 1:**
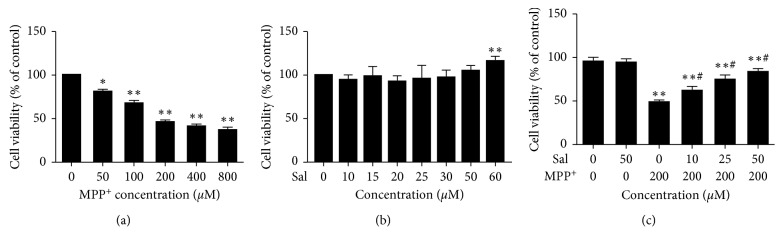
Sal ameliorated the cytotoxicity induced by MPP^+^ in MN9D cells. (a) The cytotoxicity of various concentrations of MPP^+^ on MN9D cells for 24 h. (b) The effect of various concentrations of Sal on the cell viability for 24 h. (c) The effect of pretreatment with Sal (10, 25, and 50 *μ*M) on MPP^+^-induced cytotoxicity. Each column represents the mean ± SD (*n*=3). ^*∗*^ *P* < 0.05 and ^*∗∗*^ *P* < 0.01, compared with the control group; ^#^ *P* < 0.05 and ^##^ *P* < 0.01, compared with the MPP^+^-treated group.

MN9D cells were treated with various concentrations of MPP^+^ for 24 h. Results of MTT indicated that 200 *μ*M of MPP^+^ remarkably reduced the cell viability (Figure 1(a)). To examine whether Sal alone had an effect on cell viability, we treated cells with different concentrations of Sal for 24 h. The Sal at 0–50 *μ*M had no obvious effect on cell viability (Figure 1(b)). Then, we investigated the neuroprotective effect of Sal on MPP^+^-induced cell toxicity, which was markedly prevented by Sal (10, 25, and 50 *μ*M) (Figure 1(c)). Given the results of MTT, cells were pretreated with or without Sal (10, 25, 50 *μ*M) for 24 h followed by MPP^+^ (200 *μ*M) for an additional 24 h in the following experiments.

### 3.2. Effect of Sal on MPTP-Induced Behavioral Deficits

**Figure 2:**
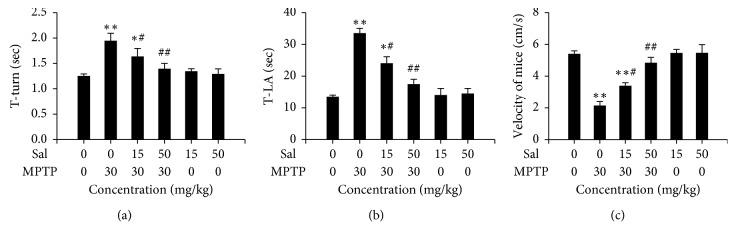
Effect of treatment with Sal (15, 50 mg/kg/day, 7 days), MPTP (30 mg/kg/day, 5 days), or their combination on behavior performance of mice. (a) The time obtained for mice to turn completely downward (T-turn). (b) The time obtained for mice to climb to the floor (T-LA). (c) The speed of mice in the wooden box. Each column represents the mean ± SD (*n*=10). ^*∗*^ *P* < 0.05 and ^*∗∗*^ *P* < 0.01, compared with the control group; ^#^ *P* < 0.05 and ^##^ *P* < 0.01, compared with the MPTP-treated group.

The results of the pole test and open field test are shown in Figure 2. In the pole test, the time to T-turn and T-LA is longer in MPTP-treated mice, compared with the control mice (Figures 2(a) and 2(b)). Next, in the open field test, the MPTP-treated mice were significantly slow in velocity of movement compared with the control mice (*P* < 0.01; Figure 2(c)). Sal treatment significantly alleviated these behavior disorders induced by MPTP (*P* < 0.05 or *P* < 0.01), and Sal alone had no apparent effect.

### 3.3. Effect of Sal on MPTP-Induced Reduction of TH-Positive Neurons

**Figure 3:**
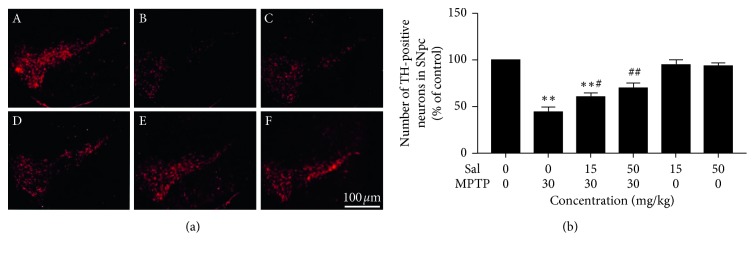
Effect of treatment with Sal, MPTP, or their combination on TH-positive neurons in the substantia nigra of mice. (a) Representative photomicrographs of TH-positive neurons in the substantia nigra. (b) Quantitative analysis of the number of TH-positive neurons in the substantia nigra. Each column represents the mean ± SD (*n*=3). ^*∗*^ *P* < 0.05 and ^*∗∗*^ *P* < 0.01, compared with the control group; ^#^ *P* < 0.05 and ^##^ *P* < 0.01, compared with the MPTP-treated group.

The results of immunofluorescent assay displayed that, in comparison with the control group, the number of TH-positive neurons in the SN of the MPTP group was significantly reduced (*P* < 0.01; Figures 3(a) and 3(b)). The number of TH-positive neurons in the mice that were treated with 15 and 50 mg/kg Sal was significantly increased in comparison with that in the MPTP group (*P* < 0.05; *P* < 0.01). Moreover, no differences in TH-positive neurons were detected in Sal alone-treated mice.

### 3.4. Effects of Sal on MPP^+^/MPTP-Induced Oxidative Stress

**Figure 4:**
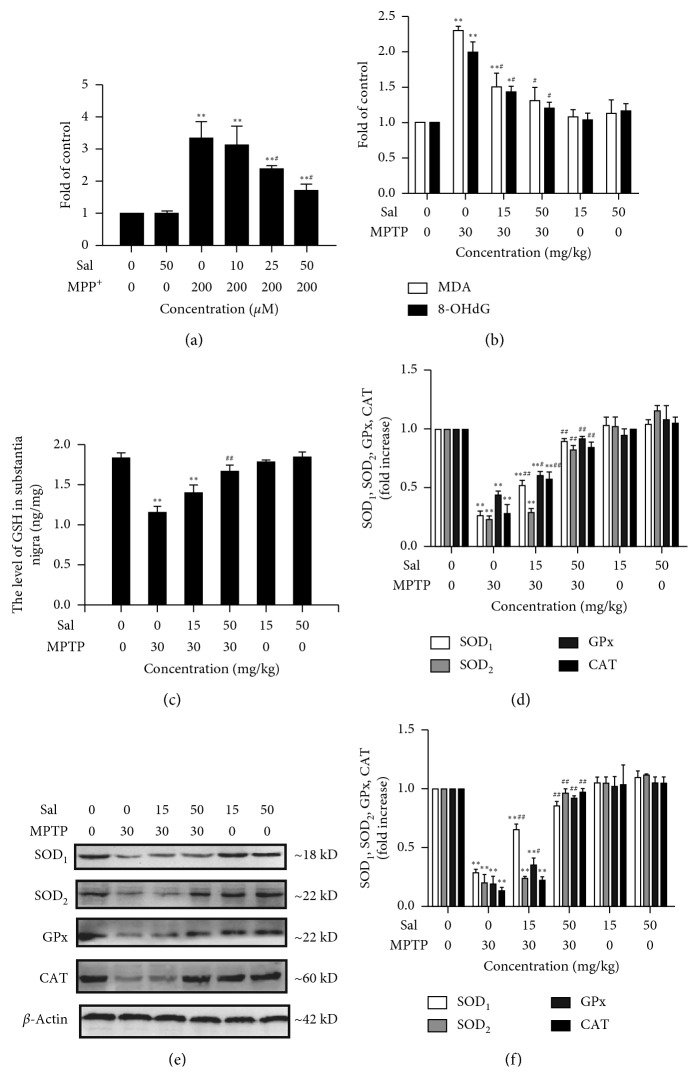
Sal inhibited oxidative stress and increased the generation of endogenous antioxidants. (a) The level of ROS in MN9D cells. (b) The level of MDA and 8-OHdG in the substantia nigra of mice. (c) The level of GSH in the substantia nigra of mice. The level (d) and the protein expression (e, f) of SOD1, SOD2, GSH-Px, and CAT in the substantia nigra of mice. Each column represents the mean ± SD (*n*=4). ^*∗*^ *P* < 0.05 and ^*∗∗*^ *P* < 0.01, compared with the control group; ^#^ *P* < 0.05 and ^##^ *P* < 0.01, compared with the MPP^+^/MPTP-treated group.

To confirm whether Sal modulated the oxidative stress, we measured the ROS, MDA, and 8-OHdG levels. As shown in Figure 4(a), MPP^+^ treatment can significantly increase the ROS levels in cells, compared with those of the control group, but pretreatment with different concentrations of Sal can abolish MPP^+^-induced overproduction of ROS (*P* < 0.05, *P* < 0.01; Figure 4(a)). Then, Sal modulation on MDA and 8-OHdG levels in the SN was investigated. As shown in Figure 4(b), the MDA and 8-OHdG levels in MPTP-treated mice significantly increased, compared with the control group (*P* < 0.01). However, the accumulation of MDA and 8-OHdG significantly reduced dose-dependently by Sal treatment (*P* < 0.05, *P* < 0.01; Figure 4(b)). Additionally, no differences were detected in Sal alone-treated mice.

### 3.5. Effect of Sal on the Levels of Endogenous Antioxidants

In the body, oxidative stress could be counteracted by endogenous and exogenous antioxidants [20]. We investigated whether Sal can enhance the content of endogenous antioxidants by measuring the GSH, SOD1, SOD2, GSH-Px, and CAT levels, as assayed by ELISA. The GSH content in the SN of MPTP-treated group significantly decreased, compared with the control group (*P* < 0.01). However, Sal treatment significantly alleviated MPTP-induced loss of GSH (Figure 4(c)). In addition, the SOD1, SOD2, GSH-Px, and CAT activities in MPTP-treated mice significantly decreased, compared with the control group (*P* < 0.01), and Sal attenuated this decrease dose-dependently. Sal alone did not show a significant effect on GSH, SOD1, SOD2, GSH-Px, and CAT levels, compared with the control group (Figure 4(d)).

Western blot revealed that the expression of SOD1, SOD2, GSH-Px, and CAT was significantly decreased by MPTP treatment, compared with the control group (*P* < 0.01; Figures 4(e) and 4(f)). Meanwhile, Sal treatment significantly inhibited the decrease in the expression of SOD1, SOD2, GSH-Px, and CAT dose-dependently. However, no differences were detected in Sal alone-treated mice.

### 3.6. Effect of Sal on the DJ-1/Nrf2 Expression *In Vitro* and *In Vivo*

**Figure 5:**
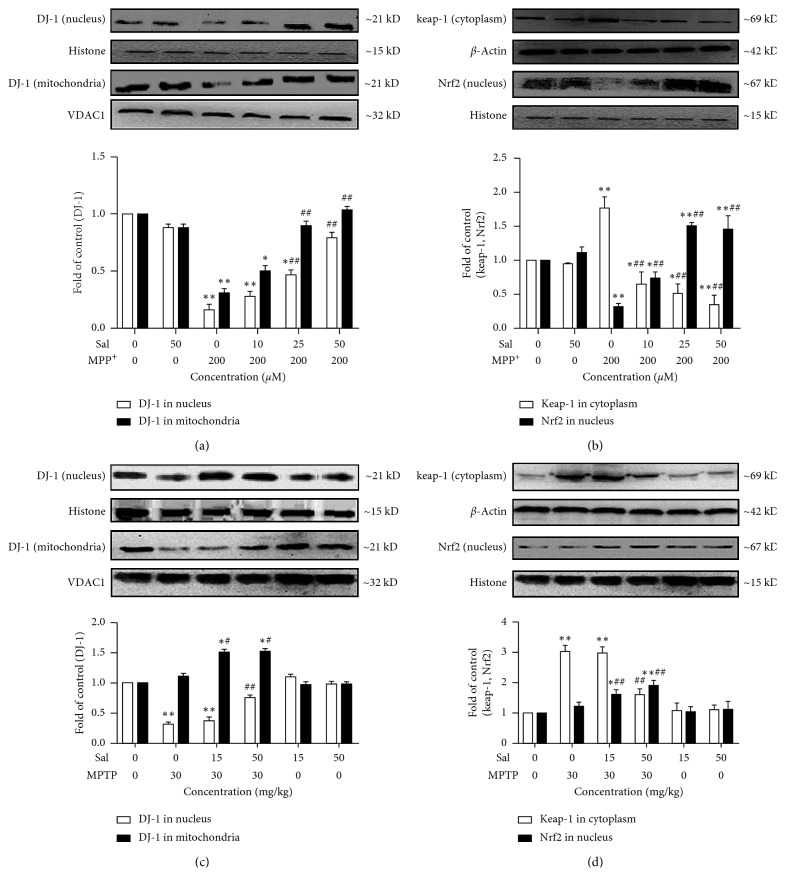
Regulation of DJ-1 and Nrf2 expression. Regulation of DJ-1 expression in MN9D cells (a) and the substantia nigra of mice (c). Regulation of Nrf2 and Keap1 expression in MN9D cells (b) and the substantia nigra of mice (d). Each column represents the mean ± SD (*n*=3). ^*∗*^ *P* < 0.05 and ^*∗∗*^ *P* < 0.01, compared with the control group; ^#^ *P* < 0.05 and ^##^ *P* < 0.01, compared with the MPP^+^/MPTP-treated group.

To determine the contribution of DJ-1/Nrf2 on the antioxidative stress effect of Sal, we first measured the protein expression of DJ-1 and Nrf2. As shown in Figure 5(a), Sal treatment increased the nuclear and mitochondrial accumulation of DJ-1 in comparison with the MPP^+^ group. Western blot results *in vivo* showed Sal treatment increased the nuclear and mitochondrial accumulation of DJ-1 in comparison with the MPTP group and no differences were detected in the Sal alone group (Figure 5(c)). Moreover, as shown in Figure 5(b), Sal treatment increased the nuclear Nrf2 and decreased the cytoplasmic expression of Keap1 in comparison with the MPP^+^ group. However, Sal alone had no obvious influence on Nrf2 and Keap1 expression. Western blot *in vivo* revealed that Sal increased the nuclear Nrf2 and decreased the cytoplasmic expression of Keap1 dose-dependently compared with MPTP-treated mice (*P* < 0.01, *P* < 0.05; Figure 5(d)). However, transfected with DJ-1/Nrf2 siRNA abrogated the inhibitory effect of Sal on ROS generation (Figure S2) and inhibited Sal-induced nuclear/mitochondrial accumulation of DJ-1 and the nuclear accumulation of Nrf2 (Figure S3). These results supported the role of DJ-1/Nrf2 on the antioxidative stress effect of Sal.

### 3.7. Effects of Sal on the Complex I Activity *in Vitro* and *in Vivo*

**Figure 6:**
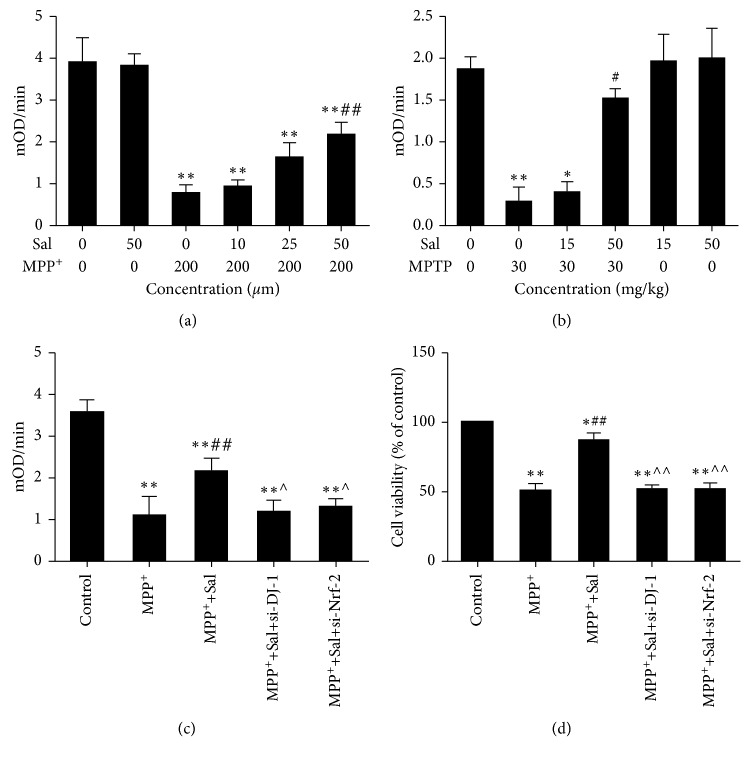
Role of DJ-1/Nrf2-mediated antioxidant effect for the maintenance of complex I activity and neuroprotective effect of Sal. Sal reversed MPP^+^/MPTP-induced impairment of complex I activity in MN9D cells (a) and the substantia nigra of mice (b). Effect of silencing DJ-1 and Nrf2 on complex I activity (c) and cell viability (d) of MN9D cells. Each column represents the mean ± SD (*n*=3). ^*∗*^ *P* < 0.05 and ^*∗∗*^ *P* < 0.01, compared with the control group; ^#^ *P* < 0.05 and ^##^ *P* < 0.01, compared with the MPP^+^/MPTP-treated group; ^˄^ *P* < 0.05 and ^˄˄^ *P* < 0.01, compared with the MPP^+^-Sal treated group.

To understand the mechanisms of the protective effects of Sal in mitochondria, we measured the complex I activities *in vitro* and *in vivo*. The complex I activity of the MPP^+^ group significantly decreased, compared with that of the control group in MN9D cells (*P* < 0.01; Figure 6(a)). Moreover, pretreatment with different concentrations of Sal can abolish MPP^+^-induced inhibition of complex I activity dose-dependently in MN9D cells. *In vivo*, complex I activity of the MPTP-treated group significantly decreased, compared with that of the control group (*P* < 0.01; Figure 6(b)). However, Sal treatment significantly reversed MPTP-induced decrease in complex I activity dose-dependently. Cells and mice treated by Sal alone had no significant difference compared with the control group *in vitro* and *in vivo*.

### 3.8. Role of DJ-1/Nrf2-Mediated Antioxidant Effect for the Maintenance of Complex I Activity and Neuroprotective Effect of Sal

To further study whether DJ-1/Nrf2-mediated antioxidant effect contributes to the maintenance of complex I activity and the neuroprotective effects of Sal, DJ-1 and Nrf2 were silenced using siRNAs (Figure S1). Data shown in Figures 6(c) and 6(d) showed that silencing of DJ-1 and Nrf2 inhibited the increase of complex I activity and cell viability elicited by Sal. These results notably indicated DJ-1/Nrf2-mediated antioxidant effects plays an essential role in the neuroprotective effects of Sal.

## 4. Discussion

In the present study, we demonstrated that Sal plays a neuroprotective effect against MPP^+^/MPTP-induced impairment of DA neurons by preserving complex I activity via DJ-1/Nrf2-mediated antioxidant pathway, as evidenced by the following: (1) Sal pretreatment significantly increased the cell viability in MN9D cells subjected to MPP^+^; (2) administration of Sal notably attenuated behavioral impairments, accompanied by decreased TH-positive neurons in the SN of the MPTP-induced PD model; (3) Sal decreased the oxidative stress and increased the levels of endogenous antioxidants; (4) Sal increased the nuclear translocation of DJ-1 and Nrf2 and the mitochondrial translocation of DJ-1, accompanied by activating complex I; (5) knockdown of DJ-1/Nrf2 expression with siRNA abolished the protective effects of Sal on complex I and cell viability in the MPP^+^-induced PD model.

Oxidative stress is characterized by ROS overproduction and enzymatic and nonenzymatic antioxidant deficiency in the biological system [21]. Excessive ROS can damage all macromolecules, including nucleic acids, lipids, and proteins, leading to an overall progressive decline in physiological function [5]. In our study, we examined the oxidative stress via measuring the ROS production and levels of the oxidative products, including lipid peroxidation product (MDA) and DNA oxidation product (8-OHdG) [22, 23]. The results indicated that Sal plays important roles in inhibiting oxidative stress, as evidenced by diminishing ROS accumulation, MDA, and 8-OHdG levels against MPP^+^/MPTP. The antioxidant systems include enzymatic scavengers, such as SOD, CAT, and GSH-Px [20]. SODs belong to the family of metalloenzymes and can scavenge highly toxic OH^−^ by catalyzing the dismutation of superoxide radicals into O_2_ and H_2_O_2_ [24]. Among the superfamily of SODs, SOD1 and SOD2 play important roles in eliminating excess ROS to protect tissues from oxidative damage [25]. SOD1 is mainly located in the cytoplasm and is one of the most important free-radical scavengers that respond to oxidative stress [26]. SOD2 is a mitochondrial antioxidant whose deficiency causes devastating effects to mitochondrial metabolism [26, 27]. In addition, CAT and GSH-Px convert H_2_O_2_ to H_2_O [20]. In our present study, the activities of GSH, SOD1, SOD2, CAT, and GSH-Px were declined in MPTP-treated mice and Sal treatment ameliorated the decline in GSH, SOD1, SOD2, CAT, and GSH-Px activities. Altogether, our findings indicated the neuroprotective effect of Sal on the MPP^+^/MPTP-induced PD model, which was associated with decreased oxidative damage and increased antioxidants.

DJ-1 is a recessively inherited Parkinson's gene and functions as an antioxidant through a variety of mechanisms, including a weak direct antioxidant activity by scavenging ROS [10]. Moreover, DJ-1 also functions as an antioxidant by upregulating the expression of other antioxidant genes, such as the transcriptional factor Nrf2, a master regulator of antioxidant gene [10]. DJ-1 is mainly localized in the cytosol and can be translocated to mitochondria and nucleus against oxidative stress-induced cell death under oxidative status [28]. Under normal resting metabolic conditions, Nrf2 is located in the cytoplasm by its antagonist Keap1 under normal conditions. Under conditions of stress, Keap1 is oxidized and releases Nrf2, which is stabilized by DJ-1, and translocates to the nucleus where, via ARE enhancers, it activates a range of antioxidant enzymes [29, 30]. In the current study, we found that Sal treatment promoted the nuclear accumulation of DJ-1 and Nrf2 and the mitochondrial accumulation of DJ-1 and inhibited Keap1 expression, compared with the MPP^+^/MPTP-treated group. To confirm the antioxidative stress effect of Sal was DJ-1/Nrf2-dependent, we silenced DJ-1/Nrf2 by using siRNA. Results showed silencing of DJ-1/Nrf2 inhibited the suppression of ROS generation and abrogated the nuclear/mitochondrial accumulation of DJ-1 and the nuclear accumulation of Nrf2 induced by Sal.

Due to the fact that oxidative stress is intimately linked to complex I dysfunction, numerous neuroprotective mechanisms converge on preserving complex I via antioxidant therapeutic strategies [31, 32]. We first investigated the effect of Sal on complex I, and results indicated that Sal effectively prevented the decrease in complex I activity against MPP^+^/MPTP. To further investigate whether DJ-1/Nrf2-mediated antioxidative stress involved in the observed effect of Sal on maintenance of complex I activity and improvement of cell injury, we silenced DJ-1 and Nrf2 by using siRNA. Our present study demonstrated silencing of DJ-1 and Nrf2 abolished the increase in complex I activity and cell viability elicited by Sal. The above data supported that maintenance of complex I activity by Sal through DJ-1/Nrf2-mediated antioxidant pathway serves as a crucial mechanism by which Sal attenuates complex I dysfunction and plays neuroprotective roles in the MPP^+^/MPTP-induced PD model.

In accordance with our previous studies, the findings support the idea that Sal exhibits potent neuroprotective effects against the MPP^+^/MPTP-induced PD model through preserving complex I activity via DJ-1/Nrf2-mediated antioxidant pathway. The neuroprotective effect of Sal may hold promise as a preventive therapy of PD.

However, our study has limitations. It is noteworthy that the expression of Nrf2 is cell-type-specific. In contrast to neuronal cell lines, there are data showing that the neuronal Nrf2 activity is low in cortical neurons due to epigenetic repression of Nrf2 gene promoter early in development [33]. The results of Nrf2 presented in our study are restricted to cell lines and are not applicable to bona fide neurons. Therefore, further studies on bona fide neurons are needed to verify the neuroprotective effect of Sal. Another limitation of our study is that, in addition to the antioxidant pathway of Nrf2 in maintaining complex I activity, it also has roles in the regulation of mitochondrial biogenesis and mitophagy. Ahuja et al. found that, in Nrf2-KO MEFs and Nrf2-KO mice, mitochondrial biogenesis was decreased, as evidenced by the decrease in the content of mtDNA, the protein expression of ETC complexes, and the genes involved in replication and transcription of mtDNA [34]. Another important process that maintains mitochondrial quality control is mitophagy. PINK1 (encoded by *PARK6* gene) interacts with Parkin (encoded with *PARK2* gene) to maintain mitochondrial membrane integrity and target dysfunctional mitochondria for autophagy. There are data showing that Nrf2 can upregulate PINK1 expression, and exogenous Nrf2 stimulation protected PINK-KO and PINK1-WT cell lines from DA-induced toxicity [35, 36]. Thus, more studies should be conducted to investigate whether Sal confers neuroprotective effects via regulating mitochondrial biogenesis and mitophagy.

## Figures and Tables

**Table 1:** siRNA sequences of MN9D cells.

Gene	Forward primer	Reverse primer
*NFE2L2*	5′-GCAAGUUUGGCAGGAGCUAdTdT-3′	5′-UAGCUCCUGCCAAACUUGCdTdT-3′
*NFE2L2*	5′-CCGAAUUACAGUGUCUUAAdTdT-3′	5′-CCGAAUUACAGUGUCUUAAdTdT-3′
*NFE2L2*	5′-GCAACUGUGGUCCACAUUUdTdT-3′	5′-AAAUGUGGACCACAGUUGCdTdT-3′
*PARK7*	5′-GCAGUGUAGCCGUGAUGUAdTdT-3′	5′-UACAUCACGGCUACACUGCdTdT-3′
*PARK7*	5′-CAGUGUAGCCGUGAUGUAAdTdT-3′	5′-UUACAUCACGGCUACACUGdTdT-3′
*PARK7*	5′-AGUGUAGCCGUGAUGUAAUdTdT-3′	5′-AUUACAUCACGGCUACACUdTdT-3′

## Data Availability

The data used to support the findings of this study are available from the corresponding author upon request.

## Supplementary Materials

Supplementary Materials Figure S1: the results of the DJ-1 and Nrf2 expression after transfecting siRNA for 72 h. Si-Nrf2 (3#, 25 nM) and si-DJ-1(3#, 100 nM) were used in the following experiments. Figure S2: silencing DJ-1 and Nrf2 abolished the inhibitory effect of Sal on ROS generation. Figure S3: silencing DJ-1 and Nrf2 inhibited Sal-induced nuclear/mitochondrial accumulation of DJ-1 and the nuclear accumulation of Nrf2. Figure S4: the gray value and quantitative analysis of the nuclear DJ-1 expression in MN9D cells. Figure S5: the gray value and quantitative analysis of the mitochondrial DJ-1 expression in MN9D cells. Figure S6: the gray value and quantitative analysis of the nuclear DJ-1 expression in the SN of mice. Figure S7: the gray value and quantitative analysis of the mitochondrial DJ-1 expression in the SN of mice. Figure S8: the gray value and quantitative analysis of the nuclear Nrf2 expression in MN9D cells. Figure S9: the gray value and quantitative analysis of the nuclear Nrf2 expression in the SN of mice.
