# Supplementary material for: Salidroside Protects Dopaminergic Neurons by Preserving Complex I Activity via DJ-1/Nrf2-Mediated Antioxidant Pathway
Source: Parkinsons Dis. 2019 May 16;2019:6073496. doi: 10.1155/2019/6073496 (PMC6541949; doi:10.1155/2019/6073496)
Supplement: Supplementary Materials — Figure S1: the results of the DJ-1 and Nrf2 expression after transfecting siRNA for 72 h. Si-Nrf2 (3#, 25 nM) and si-DJ-1(3#, 100 nM) were used in the following experiments. Figure S2: silencing DJ-1 and Nrf2 abolished the inhibitory effect of Sal on ROS generation. Figure S3: silencing DJ-1 and Nrf2 inhibited Sal-induced nuclear/mitochondrial accumulation of DJ-1 and the nuclear accumulation of Nrf2. Figure S4: the gray value and quantitative analysis of the nuclear DJ-1 expression in MN9D cells. Figure S5: the gray value and quantitative analysis of the mitochondrial DJ-1 expression in MN9D cells. Figure S6: the gray value and quantitative analysis of the nuclear DJ-1 expression in the SN of mice. Figure S7: the gray value and quantitative analysis of the mitochondrial DJ-1 expression in the SN of mice. Figure S8: the gray value and quantitative analysis of the nuclear Nrf2 expression in MN9D cells. Figure S9: the gray value and quantitative analysis of the nuclear Nrf2 expression in the SN of mice. [file 6073496.f1.zip › 6073496.f1/Supplementary File.docx]

# Supplementary Materials

# Figure S1

**Figure S1** The result of the DJ-1 and Nrf2 expression after transfecting with various concentration of siRNA for 72 h. Si-Nrf2 (3**#**, 25nM) and si-DJ-1(3**#**, 100nM) were used in the following experiments. Each column represents the mean ± SD (n=3). **P < 0.01, compared with control group

# Figure S2

**Figure S2.** Silencing DJ-1 and Nrf2 abolished the inhibitory effect of Sal on ROS generation. Each column represents the mean ± SD (n=3). *P < 0.05 and **P < 0.01, compared with the control group; #P < 0.05 and ##P < 0.01, compared with MPP^+^-treated group; ^P < 0.05 and ^^P < 0.01, compared with MPP^+^-Sal treated group.

Figure S3

**Figure S3.** Silencing DJ-1 and Nrf2 inhibited Sal-induced nuclear/mitochondrial accumulation of DJ-1 and the nuclear accumulation of Nrf2. Each column represents the mean ± SD (n=3). *P < 0.05 and **P < 0.01, compared with the control group; #P < 0.05 and ##P < 0.01, compared with MPP^+^-treated group.

Figure S4

|  |  |
| --- | --- |
|  |  |

The gray value and quantitative analysis of the nuclear DJ-1 expression in MN9D cells. Each column represents the mean ± SD (n=3).*P < 0.05, compared with control group; ##P < 0.01, compared with MPP^+^-treated group.

Figure S5

|  |  |
| --- | --- |
|  |  |

The gray value and quantitative analysis of mitochondrial DJ-1 expression in MN9D cells. Each column represents the mean ± SD (n=3).*P < 0.05 and **P < 0.01, compared with control group; ##P < 0.01, compared with MPP^+^-treated group.

Figure S6

|  |  |
| --- | --- |
|  |  |

The gray value and quantitative analysis of the nuclear DJ-1 expression in the SN of mice. Each column represents the mean ± SD (n=3). **P < 0.01, compared with control group; #P < 0.05, compared with MPTP-treated group.

**Figure S7**

|  |  |
| --- | --- |
|  |  |

The gray value and quantitative analysis of the mitochondrial DJ-1 expression in the SN of mice. Each column represents the mean ± SD (n=3). *P < 0.05, compared with control group; #P < 0.05 and ##P < 0.01, compared with MPTP-treated group.

**Figure S8**

|  |  |
| --- | --- |
|  |  |

The gray value and quantitative analysis of the nuclear Nrf2 expression in MN9D cells. Each column represents the mean ± SD (n=3).*P < 0.05, compared with control group; ##P < 0.01, compared with MPP^+^-treated group.

**Figure S9**

|  |  |
| --- | --- |

The gray value and quantitative analysis of the nuclear Nrf2 expression in the SN of mice. Each column represents the mean ± SD (n=3).*P < 0.05 and **P < 0.01, compared with control group; ##P < 0.01, compared with MPTP-treated group.
